# The prion protein regulates beta-amyloid-mediated self-renewal of neural stem cells *in vitro*

**DOI:** 10.1186/s13287-015-0067-4

**Published:** 2015-04-11

**Authors:** Steven J Collins, Carolin Tumpach, Qiao-Xin Li, Victoria Lewis, Timothy M Ryan, Blaine Roberts, Simon C Drew, Victoria A Lawson, Cathryn L Haigh

**Affiliations:** Department of Pathology, The University of Melbourne, Melbourne Brain Centre, Melbourne, VIC 3010 Australia; Department of Medicine, Royal Melbourne Hospital, The University of Melbourne, Melbourne, VIC 3010 Australia; Florey Institute of Neuroscience and Mental Health, The University of Melbourne, Melbourne, VIC 3010 Australia; The Florey Department of Neuroscience and Mental Health, The University of Melbourne, Melbourne, VIC 3010 Australia

## Abstract

The beta-amyloid (Aβ) peptide and the Aβ-oligomer receptor, prion protein (PrP), both influence neurogenesis. Using *in vitro* murine neural stem cells (NSCs), we investigated whether Aβ and PrP interact to modify neurogenesis. Aβ imparted PrP-dependent changes on NSC self-renewal, with PrP-ablated and wild-type NSCs displaying increased and decreased cell growth, respectively. In contrast, differentiation of Aβ-treated NSCs into mature cells was unaffected by PrP expression. Such marked PrP-dependent differences in NSC growth responses to Aβ provides further evidence of biologically significant interactions between these two factors and an important new insight into regulation of NSC self-renewal *in vivo*.

**Electronic supplementary material:**

The online version of this article (doi:10.1186/s13287-015-0067-4) contains supplementary material, which is available to authorized users.

## Findings

### Introduction

Alzheimer’s disease (AD) is the most common form of dementia. The core components of the senile plaques that characterise AD pathologically are beta-amyloid (Aβ) peptides cleaved from the amyloid precursor protein (APP). Various Aβ species exist as a result of differing N- and C-terminal processing sites and these species can aggregate, forming oligomers that are implicated in Aβ toxicity [1]. Most Aβ species are found in healthy brain tissue but the relative amounts shift during AD [2,3]. In health, Aβ1-40 predominates and during AD Aβ1-42, Aβ4-42 and pyroglutamated Aβ3-42 (3(pE)-42) are increased [3]. Many other species also exist with their relative amounts changing during disease.

Neurogenesis, whilst declining significantly in the adult organism, continues throughout life. Adult neural stem cells (NSCs) are confined to specific protected sites within the brain, including the sub-granular zone (SGZ) of the dentate gyrus and sub-ventricular zone of the lateral ventricle [4]. Adult NSCs can self-renew and are multipotent; they can differentiate into cells of any central nervous system lineage. In the brains of AD patients, markers of neurogenesis are increased [5,6] indicating potential neurogenic dysregulation or stimulated compensation for neuronal loss. AD pathology typically begins in the transentorhinal and entorhinal cortex [7]. This region lies adjacent to the SGZ and, therefore, NSCs in their normally protected niche environment may be exposed to hostile conditions that stimulate them to change their behaviour [4]. There is significant evidence that Aβ peptides are able to modulate neurogenesis [8-10]. Various discrepancies exist in the literature as to whether neurogenesis is enhanced or suppressed by Aβ exposure, which is most likely due to the manner in which the Aβ was prepared (that is, if monomeric, oligomeric or fibrillar Aβ species were used) and the model system for NSC study (for example, *in vivo, in vitro*, mouse strain); however, the consensus is in favour of changed NSC behaviour following exposure to Aβ species.

Neurogenesis is also modulated by another neurodegenerative disease-associated protein, the prion protein (PrP) [11,12]. Increased PrP expression is associated with increased cell cycling at the expense of differentiation [13]. Recent studies found that PrP is an essential receptor that transduces soluble Aβ1-42 oligomer signals from the plasma-membrane through the NMDA receptor via the signalling molecule fyn to tau, with this signalling thought to cause cellular toxicity [14-16]. Based on the knowledge that both Aβ and PrP can individually modulate neurogenesis and that PrP is a soluble Aβ1-42 binding partner necessary for the transduction of toxic signals, we hypothesized that PrP might also transduce the Aβ peptide signals that alter neurogenesis. The present study therefore investigated the ability of various Aβ peptides to modulate *in vitro* self-renewal and differentiation of adult NSCs harvested from PrP gene-ablated (knock-out (KO)) or from wild-type (WT; normal PrP expression) mice.

## Methods

Aβ-amyloid peptides (China Peptides, China) were prepared as described previously [17]. NSC harvest and routine culture was as described previously [18,19]. For the neural colony-forming assay, cells were seeded in a semi-solid gel matrix made with a 2:1 solution of proliferation medium and collagen. After day 21, neurospheres were counted and their diameter measured using NIS-Elements (Nikon Adelaide, Australia) software. Cell cycle analysis was performed using the Muse Cell Cycle Kit (Millipore, Bayswater, Victoria AUS). For plate and blotting assays cells were cultured as an adherent monolayer on a 1:1 poly-D-lysine-laminin matrix. Cellular ATP content was measured using Life Sciences’ ATP assay (Invitrogen, Mulgrave, Victoria AUS). Immunodetection methods have been described previously [18-20]. Expanded methodology is provided in Additional file 1.

## Results and discussion

Potential Aβ-PrP signalled changes in neurogenesis were assessed using four Aβ species; Aβ1-40, Aβ1-42, Aβ4-42 and Aβ3(pE)-42, representing those that are found ‘normally’ in health and those that have been linked with cellular toxicity in AD [21,22]. Previous studies have shown that fibrillar Aβ has no effect on neurogenesis [23] and soluble oligomeric Aβ42 is toxic; therefore, Aβ peptides were prepared using an established protocol for producing soluble monomeric species [17]. One μM Aβ peptide was used based on results of previous studies that demonstrated this concentration induced neurogenic effects [24]. No toxicity was observed at this concentration throughout the duration of the assays (Additional file 2).

**Figure 1:**
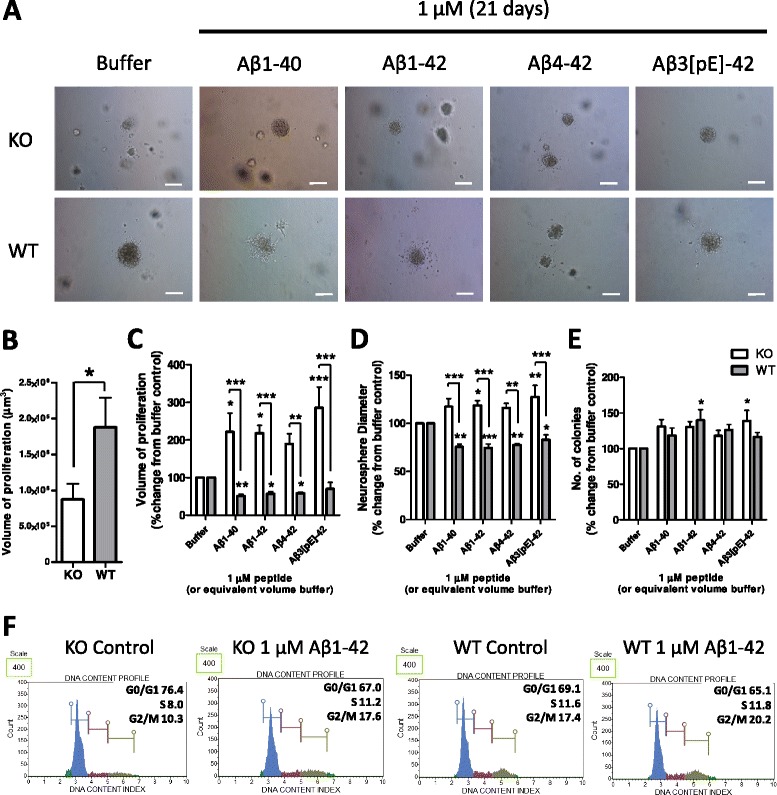
Beta-amyloid (Aβ) peptide modulation of neural stem cell (NSC) self-renewal is prion protein (PrP) dependent. **(A)** Representative images of NSC colonies grown in a collagen matrix with or without Aβ for 21 days. **(B)** Neurosphere volume of untreated knock-out (KO) and wild-type (WT) cells. **(C)** Neurosphere volume following Aβ treatment calculated from colony diameter **(D)** and number **(E)**. **(F)** Flow cytometry cell cycle phase analysis by DNA content; an example experiment from four repeats is shown. Scale bars = 100 μm. Data are represented as mean ± SEM. *P < 0.05, **P < 0.01, ***P < 0.001.

NSCs are defined by their properties of self-renewal and differentiation into mature cells of any central nervous system lineage. First we assessed the role of PrP in modifying NSC self-renewal using a neural colony-forming assay, which considers both the number of cells able to form new clonal colonies and the size of the colonies formed as an indication of clonal growth rate. As previously reported, PrP expression positively influenced NSC self-renewal (two-way analysis of variance (ANOVA), F = 7.84, *P* = 0.006, n = 4; Figure 1A & B) [11-13]. To determine whether PrP has a role in transducing Aβ-mediated changes in NSC growth, WT and KO NSCs were incubated with the Aβ species described above. The KO cells showed a significantly greater proliferation volume when compared with the carrier buffer alone and equivalently treated WT NSCs for all Aβ species (two-way ANOVA, F = 53.75, *P* < 0.001, n = 4, see Additional file 3 for complete statistical analyses; Figure 1A & C). The difference was primarily due to increased diameter of the Aβ-treated KO colonies (Figure 1D) in contrast to a decreased diameter of the WT NSCs exposed to Aβ (two-way ANOVA, F = 63.43, *P* < 0.001, n = 4), as the number of colonies formed was not significantly different between the WT and KO NSCs (Figure 1E). Proliferation of Tga20 (PrP overexpressing) NSCs treated with Aβ showed similar changes to those observed for WT cells, with no apparent PrP dose-effect at the concentration of Aβ used (Additional file 4). The presence of PrP did not confer a greater susceptibility to any one species of Aβ, indicating that neither the N- nor C-terminal truncation of Aβ is important for induction of growth changes or interaction with PrP but that the core of the peptide appears sufficient.

To further assess differences in NSC growth, Aβ1-42 was used to assess the number of cells in each phase of the cell cycle 24 hours post-Aβ addition to liquid culture. More KO cells rested in G0/G1 basally than WT (two-way ANOVA, F = 22.99, *P* = 0.003, n = 4) but after treatment with Aβ the number of KO cells actively cycling (S, G2/M phases) was significantly increased (two-way ANOVA for S phase, F = 39.18, *P* = 0.003, n = 4; for G2/M phases, F = 24.91, *P* = 0.003, n = 4; Figure 1F). There was no significant change for the WT cells, suggesting that the changes that slow the growth and reduce the diameter of neurospheres may occur after a longer period of exposure to Aβ.

**Figure 2:**
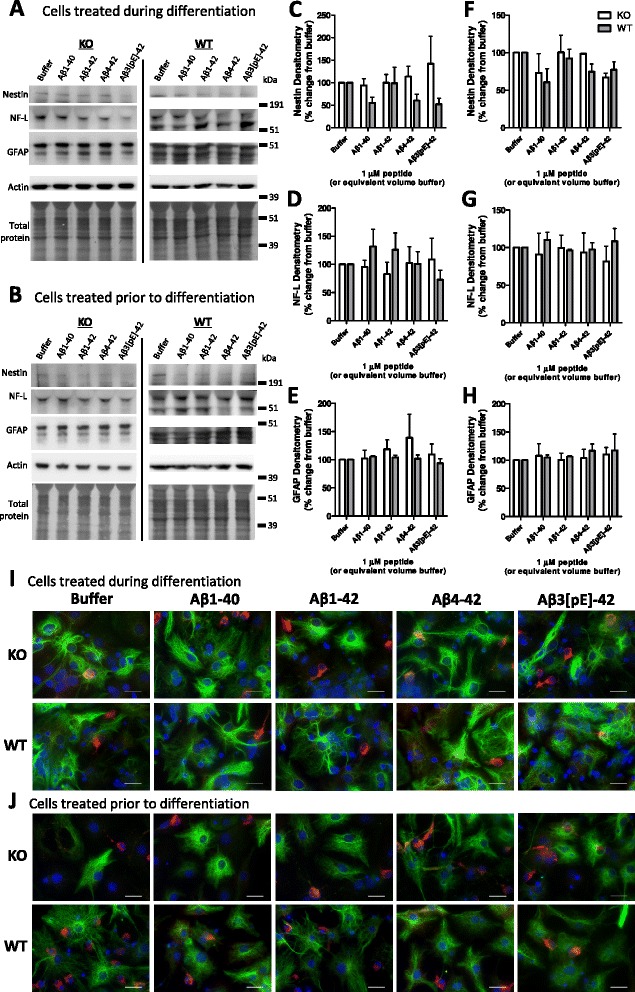
Prion protein (PrP) expression has minimal influence on how beta-amyloid (Aβ) affects neural stem cell (NSC) differentiation. Example plates of western blots for lineage protein markers from knock-out (KO) and wild-type (WT) NSCs treated for either 7 days during differentiation **(A)** or 24 hours prior to differentiation for 7 days **(B)** with 1 μM Aβ. Quantification of the western blots **(A & B)** for the stem cell marker nestin **(C, F)**, mature neuronal marker, NF-L **(D, G)**, and GFAP astrocytic marker **(E, H)**. Data are represented as mean ± SEM. Example immunofluorescent images of NSCs treated with Aβ during **(I)** and before **(J)** differentiation with NF-L, GFAP and DAPI nuclear staining shown in red, green and blue respectively. Scale bars = 15 μm.

NSCs can be induced to differentiate into neurones, astrocytes and oligodendrocytes. The lineage to which an individual cell commits is influenced by its extracellular environment both before and during stimulation to differentiate. Therefore, NSCs can be manipulated to differentiate into a specific mature cell by their environment. To assess the influence of Aβ-PrP signalling on NSC differentiation, KO and WT NSCs were either exposed to the Aβ species for 7 days (one treatment only) whilst induced to differentiate or were exposed to the Aβ species for 24 hours before induction of differentiation as an Aβ-priming event. When the effect of Aβ on WT and KO cellular differentiation was considered, no significant changes were observed in lineage preference regardless of whether Aβ was present during differentiation (two-way ANOVA for nestin, F = 4.707, *P* = 0.043 (no individually significant condition), n = 3; for NF-L, F = 1.597, *P* = 0.226, n = 3; for GFAP, F = 1.514, *P* = 0.223, n = 3; Figure 2A,C-E) or if the cells were primed by treatment before differentiation (two-way ANOVA for nestin, F = 0.616, *P* = 0.442, n = 3; for NF-L, F = 0.816, *P* = 0.377, n = 3; for GFAP, F = 0.266, *P* = 0.611, n = 3; Figure 2B,F-H; Additional file 5). However some morphological changes in WT astrocytes treated with Aβ1-42, Aβ4-42 and Aβ3(pE)-42 were observed when cells were treated during, but not when exposed before, differentiation, indicating an influence of the C-terminal two residues (Figure 2I,J). The lack of effect on lineage but morphologic change of WT cells throughout 7 days of Aβ treatment may indicate that developing astrocytic maturity and the changes in cellular protein expression associated may be required for activation of Aβx-42 signalling pathways.

**Figure 3:**
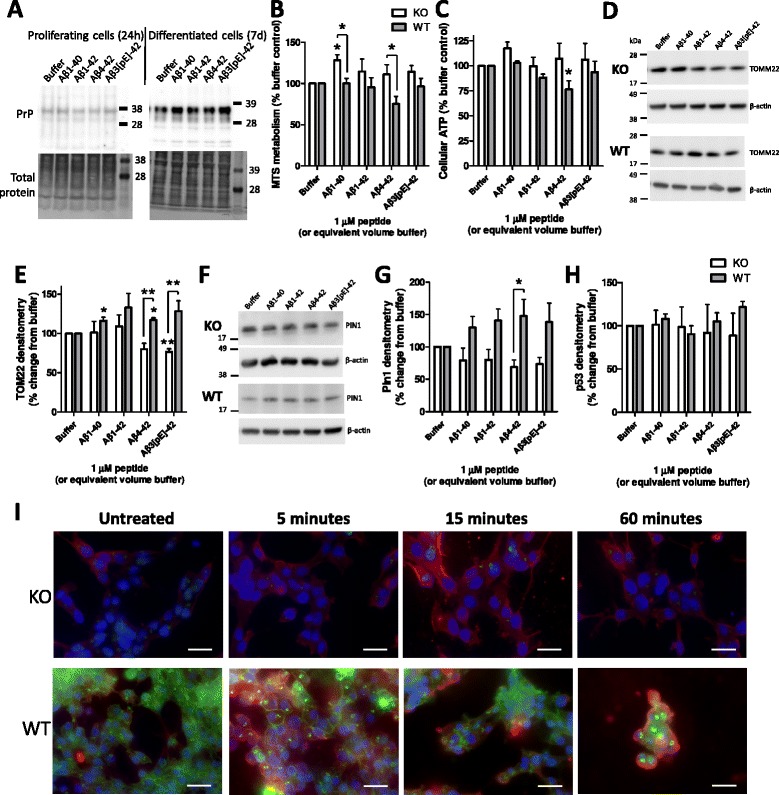
Beta-amyloid (Aβ) peptide influences on mitochondria and cell metabolism. **(A)** PrP expression in wild-type (WT) cells detected with Saf32 in the proliferating cells treated for 24 hours and in differentiated cells treated for 7 days. **(B-I)** Proliferating cells were treated for 24 hours with 1 μM of each of the Aβ species. **(B)** Cell metabolism as determined by MTS. **(C)** Cellular ATP content. **(D)** Western blots for the mitochondrial outer membrane translocase protein TOMM22. **(E)** Densitometry of the TOMM22 detection in Aβ-treated cells. **(F)** Western blots for the cell cycle protein Pin1. **(G)** Densitometry of Pin1 western blots. **(H)** Densitometry of p53 western blots. Data are represented as mean ± SEM. *P < 0.05, **P < 0.01. **(I)** Immunofluorescent images of Aβ1-42 (1 μM; WO2 red) and PrP (03R19 green) incubation with knock-out (KO) and WT neural stem cells for 0 to 60 minutes. Background red staining is endogenous amyloid precursor protein reactivity (endogenous Aβ cannot be detected in these cells; data not shown). DAPI nuclear staining is shown in blue, Scale bars = 25 μm.

With the most striking Aβ observations relating to opposing changes in self-renewal of the KO and WT cells, it was next considered how these effects might be transduced. Contrary to previous reports [25,26], in the time frames examined no significant influences of Aβ on PrP expression levels were measurable (Figure 3A; Additional file 6). Whilst chronic exposure may reduce expression over time, in the context of the experiments performed, PrP was present to perform its function.

Changes in the rate of cell cycling are likely to require energy. Therefore, markers of cellular metabolism and mitochondrial function, as well as cell cycle, were compared in WT and KO NSCs following Aβ treatment. Formazan metabolism was significantly increased in NSC KO cells compared with the WT cells when cells were treated with Aβ1-40 and Aβ4-42 (two-way ANOVA, F = 16.55, *P* < 0.001, n = 3, see Additional file 5 for basal data and Additional file 7 for full statistical analyses; Figure 3B). Cellular ATP levels were generally unchanged between the KO and WT NSCs, only showing a decrease in WT cells treated with Aβ4-42 (two-way ANOVA, F = 5.95, *P* = 0.021, n = 4; Figure 3C). This steady-state ATP measurement does not preclude increased production balanced by increased use. Contrary to decreased growth, TOMM22, a mitochondrial outer membrane translocase, was increased in WT cells when treated with Aβ1-40, Aβ4-42, and Aβ3(pE)-42 (two-way ANOVA, F = 16.45, *P* < 0.001, n = 3; Figure 3D,E). Potentially, this might indicate that Aβ can exert an effect on mitochondria signalled through PrP that could be detrimental to their function, thus limiting growth. The cell cycle marker Pin1 has also been shown to protect against tau hyperphosphorylation and subsequent changes to the cellular cytoskeleton [27]. Pin1 was globally increased in the Aβ-treated WT compared with the KO cells, although only the Aβ4-42 condition was individually significant (two-way ANOVA, F = 21.98, *P* < 0.001, n = 4; Figure 3F,G). The overall increase in Pin1 could reflect a failed effort to increase cell cycling in these cells or might represent a cellular protective response against Aβ. p53 is linked with cell cycle and also with cell death. When activated, the half-life of this protein increases resulting in increased protein detection over time. No changes in p53 protein were observed upon treatment with Aβ in either the KO or WT NSCs (Figure 3H). We additionally considered other previously PrP-linked Aβ signalling pathways (fyn, GSK-3β and calcium) finding no changes in Aβ-induced responses that relate to PrP expression (Additional file 8).

When the localisation of Aβ added to cells was considered relative to PrP, surface staining of Aβ was observed to be more intense on the WT cells after 1 hour and PrP surface staining appeared less with greater signal inside the cells (Figure 3I). These findings are consistent with previous studies that have shown Aβ preferentially binds to cells expressing PrP causing internalisation [28] and also supports the hypothesis that Aβ stimulates different pathways in KO and WT cells.

It remains to be determined how NSCs are affected by PrP-linked Aβ signalling *in vivo*, where the context of their support cells and scaffold may lead to more diverse outcomes. However, the clear responses of the cells cultured *in vitro* indicates that sufficient cellular machinery and environmental factors to transduce PrP-Aβ signalling cascades are present. The details of these pathways will be revealed by future investigation, but it is of interest that the classical Aβ signalling pathways evaluated here were unaffected. Hypothetically, as PrP-Aβ studies to date were performed in neuronal cultures or mice, different pathways could be engaged in NSCs and post-mitotic neurones or, alternatively, differing pathways may be activated when Aβ levels reach toxic concentrations. Furthermore, during sporadic AD, brain PrP expression reduces [26], inversely correlating with Aβ burden. Reduced PrP expression (akin to KO NSCs) may permit Aβ stimulation of NSC proliferation, resulting in the increased neurogenic markers seen in AD brain tissue [5,6].

## Conclusions

**Figure 4:**
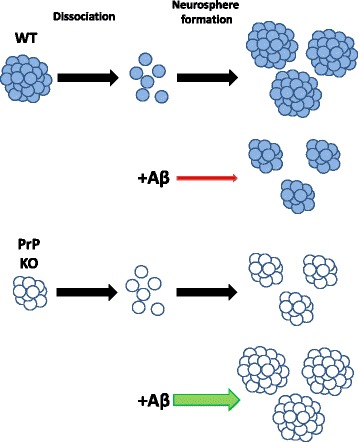
Schematic of beta-amyloid (Aβ) peptide influences on prion protein (PrP) knock-out (KO) and wild-type (WT) neural stem cell (NSC) growth. Representation of NSC neurosphere formation without Aβ and when Aβ is added to KO and WT NSCs.

This study has validated the hypothesis that the previously observed, Aβ-signalled changes in neurogenesis can be transduced through the Aβ-oligomer receptor, PrP. In addition, we demonstrated that this signalling is more complex than PrP acting as a simple ‘on’ or ‘off’ switch for the pathway (summarised in Figure 4). Our data indicate that: i) congruous with studies using neuronal cells and mice [16,29], differing Aβ pathways are activated in the NSCs depending upon the presence and absence of PrP; ii) Aβ-PrP signalling inhibits NSC proliferation signals, although possibly monomeric forms of Aβ act in contrast to the synaptotoxic soluble oligomers; and iii) variations in the basal expression levels of PrP might also account for some of the previous variability in, and disagreements over, NSC growth responses to Aβ. This dynamic and complex interplay of key factors regulating NSC growth provides significant new insight into the control of NSC self-renewal and further evidence of biologically significant interactions between Aβ peptides and PrP.

## Additional files

Additional file 1:
**Expanded methods.**
Additional file 2:
**Aβ toxicity.**
Additional file 3:
**Statistical analyses of data presented in Figure** 1 **.**
Additional file 4:
**NCFA results comparing Tga20 with KO and WT NSCs and baseline data.**
Additional file 5:
**Baseline comparisons of KO and WT cells.**
Additional file 6:
**PrP expression level changes in WT cells treated with Aβ.**
Additional file 7:
**Statistical analyses of data presented in Figure** 3 **.**
Additional file 8:
**Further signalling pathways examined in cells treated with Aβ.**

## Abbreviations

Aβ: beta-amyloid
Aβ3(pE)-42: pyroglutamated Aβ3-42
AD: Alzheimer’s disease
ANOVA: analysis of variance
KO: knock-out
NSC: neural stem cell
PrP: prion protein
SGZ: subgranular zone
WT: wild type
