# Supplementary material for: The prion protein regulates beta-amyloid-mediated self-renewal of neural stem cells in vitro
Source: Stem Cell Res Ther. 2015 Apr 11;6(1):60. doi: 10.1186/s13287-015-0067-4 (PMC4435829; doi:10.1186/s13287-015-0067-4)
Supplement: Additional file 1: — Expanded methods. [file 13287_2015_67_MOESM1_ESM.docx]

**Additional File 1: Expanded Methods.**

*β-amyloid peptides*

Peptides (95% purity) were purchased from China Peptides (China).

*β-amyloid peptides preparation*

Aβ was prepared fresh, immediately before each treatment, according to previously published protocols for generating soluble monomeric species [[1](#_ENREF_1)]. The only variations to this protocol were omission of the sonication and filtration steps.

*Adult NSC extraction*

NSCs were harvested from the sub-granular zone of PrP KO, WT and Tga20 (PrP overexpressing by approximately 8 fold) mice and transferred into suspension culture as described previously [[2](#_ENREF_2), [3](#_ENREF_3)]. All animal procedures were carried out in accordance with the NHMRC of Australia guidelines and were approved by the University of Melbourne Animal Experimentation Ethics Committee.

*NSC culture*

Routine NSC culture has been described previously [[2](#_ENREF_2)]. In brief, cells are grown as neurospheres in complete proliferation medium (Stem Cell Technologies, VIC, AUS), supplemented with 10 ng/ml FGF and 20 ng/ml EFG cytokines and 2 μg/ml heparin. Cells were maintained at 37°C in a 5% CO_2_ humidified incubator. Cells were passaged every 6-7 days by mechanical dissociation and re-seeded at 1.5 x10^6^ cells/T75 flask (uncoated).

*NSC differentiation*

Differentiation of NSCs can be induced by removal of growth factors from the cell media and addition of serum. To differentiate NSC cultures, cells were transferred into complete differentiation medium (Stem Cell Technologies) and grown on a 5 μg/ml laminin/ 50 μg/ml poly-D lysine (PDL) matrix, under normal incubator conditions, for 7 days.

*Neural colony forming assay*

Twenty-five thousand cells were seeded per condition in a 2:1 solution of proliferation medium and collagen matrix. Sixty μl of proliferation medium was added to each well every 5 days. On day 21, neurospheres were imaged using a Nikon TE3000 microscope and colonies per field were counted, with diameter measured using NIS-Elements (Nikon) software. To ensure measurement accuracy, only non-adherent and non-surface-contacted whole spheres were measured from circumference boundaries, excluding microspikes or outgrowths. Total volume was approximated using $v=n\frac{4}{3}\pi r^{3}$, where *v* = volume, *n* = number of colonies and *r* = colony radius. For clarity when comparing relative changes, within each individual repeat test conditions were normalised to the buffer control and expressed as a percentage using the formula $\%= \frac{Test}{Control}\times100$. The repeats were averaged and the mean values are shown in the graphs.

*Cell Cycle analysis*

5 × 10^5^ - 1 × 10^6^ cells were cultured with 1 μM Aβ_1-42_ (or under control conditions) for 24 hours. Cells were collected by centrifugation at 300 × g for 5 mins and mechanically dissociated in PBS before passing through a 40 μm cell strainer and centrifuging again at 300 × g. Cells were then fixed, stained and assayed using a Muse mini-flow system (Millipore, AUS) as per the Muse Cell Cycle Kit users guide (Millipore).

*Immunofluorescence staining*

Cells were seeded on a 5 μg/ml laminin/ 50 μg/ml PDL matrix in 8-well chambered coverslips at 8 x 10^5^ cells/well. The protocol for immunofluorescence staining has been described previously [[3](#_ENREF_3)]. Primary antibodies were used as follows: mouse anti-neurofilament-L (NF-L; Invitrogen) 1 in 50, rabbit anti-glial fibrilliary acidic protein (GFAP; Stem Cell Technologies) 1 in 100, WO2 at 1 in 250 [[4](#_ENREF_4)] and 03R19 [[5](#_ENREF_5)] at 1 in 2000, with 1 in 250 dilutions of the secondary antibodies.

*MTS metabolism assay*

Cells were seeded at a density of 3.6 × 10^4^ cells/well in 5 μg/ml laminin/ 50 μg/ml PDL coated 96-well plates and incubated under normal incubator conditions for the duration of the assay. Five μl one-solution MTS reagent (Promega) was added per well. Developed colour was measured at 492 nm in a SpectroSTAR UV-Vis spectrophotometer (BMG Labtech). The data were normalised and expressed as percentages as described for the NCFA.

*ATP assay*

Cells were seeded in 96-well plates as above and lysed in 20 μl RIPA buffer. Cellular ATP content was measured by luminescence using Life Sciences ATP assay (Invitrogen, VIC, AUS) as per the manufacturer’s protocol. Luminescence was measured in a PolarSTAR Optima (BMG Labtech). The data were normalised and expressed as percentages as described for the NCFA.

*Calcium assay*

Cells were seeded in 96-well plates as above. At the beginning of the assay 50 μl of culture media was removed from each well and replaced with calcium assay working buffer as per manufacturer's instructions (Invitrogen). Plates were incubated for one hour under normal incubator conditions protected from light before readings were taken using 488 nm excitation and 530 nm emission filters in a FluoSTAR Optima (BMG Labtech). The data were normalised and expressed as percentages as described for the NCFA.

*Western blotting*

Cells were seeded in 96-well plates as above. Post-treatment lysis, electrophoresis and western blotting was carried out as described previously [[2](#_ENREF_2)] with the following changes; 15-well 4-12% NuPAGE Bis-Tris gels (Invitrogen) were used and high molecular weight targets were separated using MOPS running buffer (Invitrogen). Densitometry was performed using ImageJ 1.47v and normalised to the total protein loaded (also shown by Coomassie stain [[3](#_ENREF_3)] in the differentiation experiments where the process of differentiation might change the expression levels of the standard protein markers used for loading). The data were normalised and expressed as percentages as described for the NCFA. For the differentiation experiments the same membrane was used for all four antibodies with membrane stripping and blocking between probes [[6](#_ENREF_6)]. Antibody sources and concentrations can be found below.

Antibody information for western blotting. ms = mouse, rb = rabbit.

| **Target** | **Antibody name & company** | **Concentration** | **Secondary antibody concentration** |
| --- | --- | --- | --- |
| PrP | Saf32 (ms epitope 51-90; Cayman Chemical) | 1 in 5,000 | Ms 1 in 10,000 |
| Nestin | Nestin (Sigma-Aldrich) | 1 in 1,000 | Rb 1 in 5,000 |
| Neurofilament-L | NF-L (Invitrogen) | 1 in 1,000 | Ms 1 in 2,000 |
| Glial fibrilliary acidic protein | GFAP (Stem Cell Technologies) | 1 in 5,000 | Rb 1 in 5,000 |
| p53 | p53 (Abcam) | 1 in 1,000 | Ms 1 in 2,000 |
| TOMM22 | TOMM22 (Abcam) | 1 in 1,000 | Ms 1 in 5,000 |
| Pin1 | Pin1 (Invitrogen) | 1 in 1,000 | Ms 1 in 2,000 |
| Fyn | Fyn (Abcam) | 1 in 1,000 | Rb 1 in 2,000 |
| pfyn | Pfyn (Abcam) | 1 in 1,000 | Rb 1 in 2,000 |
| GSK3b | GSK3b (Cell Signaling Technologies) | 1 in 1,1000 | Ms 1 in 2,000 |
| pGSK3b | GSK3b_ser9 (Cell Signaling Technologies) | 1 in 1,000 | Rb 1 in 2,000 |
| Actin | Actin (Sigma-Aldrich) | 1 in 5,000 | Ms 1 in 10,000 |

*Statistical analysis*

All graphs show the mean and standard error of the mean of “n” independent repeats. Statistical tests used are shown in Supplemental Tables S4-6. For one-way ANOVA, Tukeys secondary test was applied and for two-way ANOVA, Bonferroni’s secondary testing was used.

**References**

1. McColl G, Roberts BR, Gunn AP, Perez KA, Tew DJ, Masters CL, Barnham KJ, Cherny RA, Bush AI: **The Caenorhabditis elegans A beta 1-42 model of Alzheimer disease predominantly expresses A beta 3-42.** *J Biol Chem* 2009, **284:**22697-22702.

2. Haigh CL, McGlade AR, Lewis V, Masters CL, Lawson VA, Collins SJ: **Acute exposure to prion infection induces transient oxidative stress progressing to be cumulatively deleterious with chronic propagation in vitro.** *Free Radic Biol Med* 2011, **51:**594-608.

3. Sinclair L, Lewis V, Collins SJ, Haigh CL: **Cytosolic caspases mediate mislocalised SOD2 depletion in an in vitro model of chronic prion infection.** *Dis Model Mech* 2013, **6:**952-963.

4. Ida N, Hartmann T, Pantel J, Schroder J, Zerfass R, Forstl H, Sandbrink R, Masters CL, Beyreuther K: **Analysis of heterogeneous A4 peptides in human cerebrospinal fluid and blood by a newly developed sensitive Western blot assay.** *J Biol Chem* 1996, **271:**22908-22914.

5. Lawson VA, Vella LJ, Stewart JD, Sharples RA, Klemm H, Machalek DM, Masters CL, Cappai R, Collins SJ, Hill AF: **Mouse-adapted sporadic human Creutzfeldt-Jakob disease prions propagate in cell culture.** *Int J Biochem Cell Biol* 2008, **40:**2793-2801.

6. Haigh CL, Lewis VA, Vella LJ, Masters CL, Hill AF, Lawson VA, Collins SJ: **PrPC-related signal transduction is influenced by copper, membrane integrity and the alpha cleavage site.** *Cell Res* 2009, **19:**1062-1078.
