# Supplementary material for: The prion protein regulates beta-amyloid-mediated self-renewal of neural stem cells in vitro
Source: Stem Cell Res Ther. 2015 Apr 11;6(1):60. doi: 10.1186/s13287-015-0067-4 (PMC4435829; doi:10.1186/s13287-015-0067-4)

Additional file 2: Aβ toxicity.

A. Morphology of KO and WT NSCs treated for 24 hours with 1 μM Aβ. KO and WT cells have a differing morphology but no overt Aβ changes were observed. Scale bar = 20 μm. **B**. Cells were plated on a laminin/PDL matrix and treated for 7 days with a doubling dilution of Aβ1-42 in proliferation media. On day seven, cells were assayed for metabolsim using prestoblue dye (Invitrogen) as per the product protocol. Following metabolism assay the cells were lysed and LDH was assayed in media (cytotoxicity) and in the cells using a Roche LDH release assay as per the product protocol. n = 2.

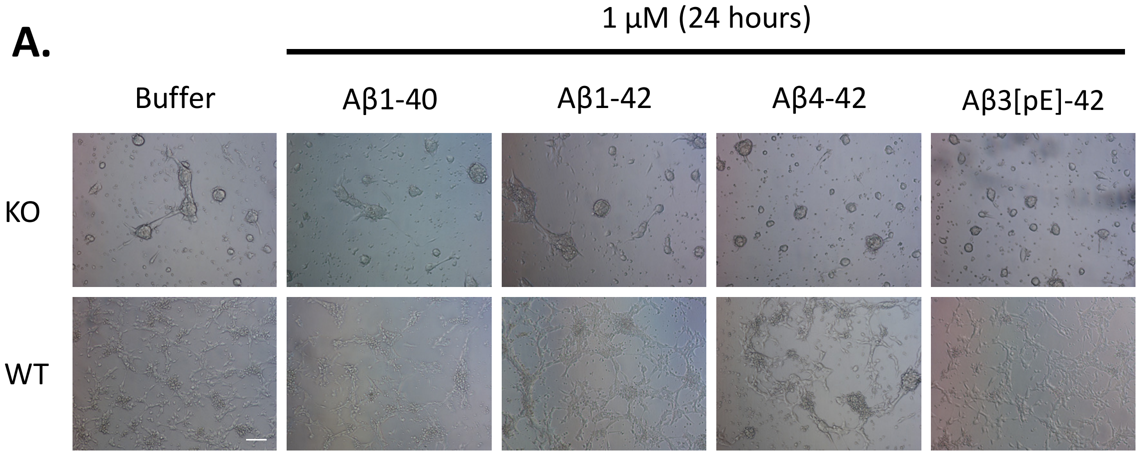

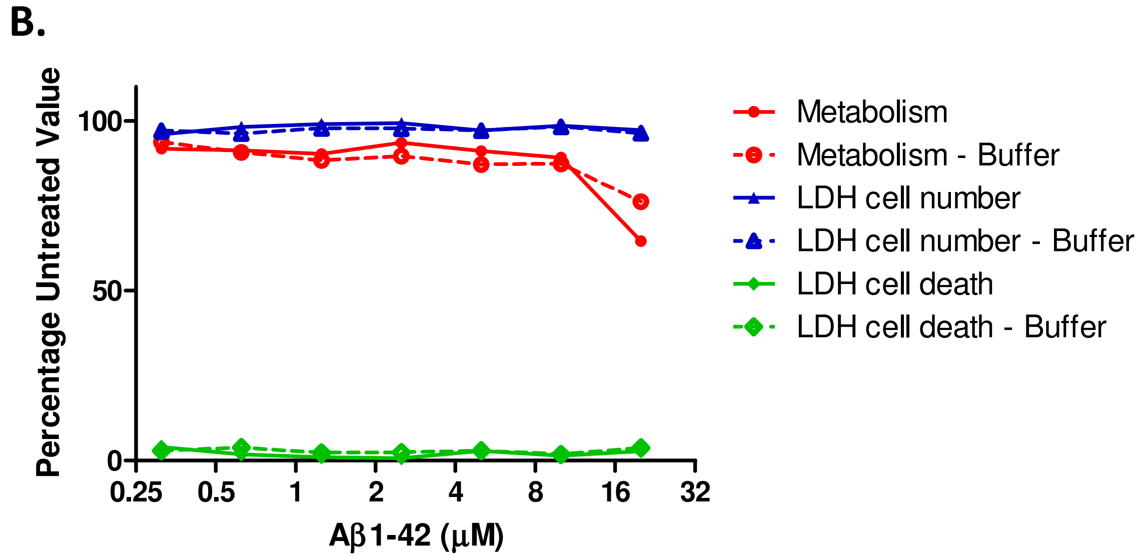

Supplement: Additional file 2: — Aβ toxicity. [file 13287_2015_67_MOESM2_ESM.pdf]
