# Supplementary material for: The prion protein regulates beta-amyloid-mediated self-renewal of neural stem cells in vitro
Source: Stem Cell Res Ther. 2015 Apr 11;6(1):60. doi: 10.1186/s13287-015-0067-4 (PMC4435829; doi:10.1186/s13287-015-0067-4)
Supplement: Additional file 3: — Statistical analyses of data presented in Figure 1 . [file 13287_2015_67_MOESM3_ESM.docx]

**Additional file 3: Statistical analyses of Aβ data presented in Figure 1 and additional file 4.**

ns = not significant, TG = Tga20.

| **Test** | **Comparison** | **Aβ1-40** | **Aβ1-42** | **Aβ4-42** | **Aβ3[pE]-42** |
| --- | --- | --- | --- | --- | --- |
| Neurosphere volume - Fig 1B  2-way ANOVA, F = 53.75, p < 0.001, n = 4 | KO Aβ vs buffer  WT Aβ vs buffer  KO vs WT  TG Aβ vs Buffer  KO vs TG  WT vs TG | P < 0.05  P < 0.01  P < 0.001  ns  P < 0.001  Ns | P < 0.05  P < 0.05  P < 0.001  ns  P < 0.001  ns | ns  P < 0.05  P < 0.01  ns  P < 0.05  ns | P < 0.001  ns  P < 0.001  P < 0.05  P < 0.001  ns |
| Neurosphere diameter - Fig 1C  2-way ANOVA, F = 63.43, p < 0.001, n = 4 | KO Aβ vs buffer  WT Aβ vs buffer  KO vs WT  TG Aβ vs Buffer  KO vs TG  WT vs TG | ns  P < 0.01  P < 0.001  P < 0.001  P < 0.001  Ns | P < 0.05  P < 0.001  P < 0.001  P < 0.001  P < 0.001  ns | ns  P < 0.01  P < 0.01  P < 0.001  P < 0.001  ns | P < 0.01  P < 0.05  P < 0.001  P < 0.001  P < 0.001  ns |
| Neurosphere colonies - Fig 1D  2-way ANOVA, F = 9.647, p < 0.001, n = 4 | KO Aβ vs buffer  WT Aβ vs buffer  KO vs WT  TG Aβ vs Buffer  KO vs TG  WT vs TG | ns  ns  ns  P < 0.05  ns  ns | ns  P < 0.05  ns  P < 0.001  ns  ns | ns  ns  ns  P < 0.001  P < 0.001  P < 0.01 | P < 0.05  ns  ns  P < 0.05  P < 0.05  ns |
| Cell cycle G0/G1 phase - Fig 1F  2-way ANOVA, F = 22.99, p = 0.003, n = 4 | KO Aβ vs buffer  WT Aβ vs buffer  KO vs WT | --  --  -- | P < 0.05  ns  P < 0.05 | --  --  -- | --  --  -- |
| Cell cycle S phase - Fig 1F  2-way ANOVA, F = 39.18, p = 0.0033, n = 4 | KO Aβ vs buffer  WT Aβ vs buffer  KO vs WT | --  --  -- | ns  ns  P < 0.01 | --  --  -- | --  --  -- |
| Cell cycle G2/M phase - Fig 1F  2-way ANOVA, F = 24.91, p = 0.0025, n = 4 | KO Aβ vs buffer  WT Aβ vs buffer  KO vs WT | --  --  -- | P < 0.05  ns  P < 0.01 | --  --  -- | --  --  -- |
| **Test** | **Comparison** | | **Result** | | |
| Neurosphere volume  2-way ANOVA, F = 7.48, p = 0.006, n = 4 | KO vs WT (untreated)  KO vs TG (untreated)  WT vs TG (untreated)  KO untreated vs buffer  WT untreated vs buffer  TG untreated vs buffer | | P < 0.05  P < 0.05  ns  ns  ns  ns | | |
| Neurosphere diameter  2-way ANOVA, F = 10.01, p = 0.003, n = 4 | KO vs WT (untreated)  KO vs TG (untreated)  WT vs TG (untreated)  KO untreated vs buffer  WT untreated vs buffer  TG untreated vs buffer | | P < 0.05  ns  ns  ns  ns  ns | | |
| Neurosphere colonies  2-way ANOVA, F = 22.13, p < 0.001, n = 4 | KO vs WT (untreated)  KO vs TG (untreated)  WT vs TG (untreated)  KO untreated vs buffer  WT untreated vs buffer  TG untreated vs buffer | | P < 0.01  ns  P <0.001  ns  ns  ns | | |
