# Supplementary material for: The prion protein regulates beta-amyloid-mediated self-renewal of neural stem cells in vitro
Source: Stem Cell Res Ther. 2015 Apr 11;6(1):60. doi: 10.1186/s13287-015-0067-4 (PMC4435829; doi:10.1186/s13287-015-0067-4)
Supplement: Additional file 4: — NCFA results comparing Tga20 with KO and WT NSCs and baseline data. [file 13287_2015_67_MOESM4_ESM.pdf]

Additional file 4: Baseline comparisons of KO and WT cells.

Quantification of basal KO and WT signal from NSCs differentiated for seven days (**A-C**) and NSCs incubated for 24 hours on the laminin/PDL matrix in proliferation media before differentiation for seven days (**D-F**) when western blotted for nestin (**A, D**), NF-L (**B, E**) or GFAP (**C, F**). Baseline rates of calcium influx (**G**), MTS metabolism (**H**) and detection of cellular ATP levels (**I**). Quantification of the basal signal from KO and WT NSCs western blotted for TOMM22 (**J**), Pin1 (**K**) and p53 (**L**). All western blot quantifications are expressed as band densitometry relative to the coomassie protein stain for protein loading.

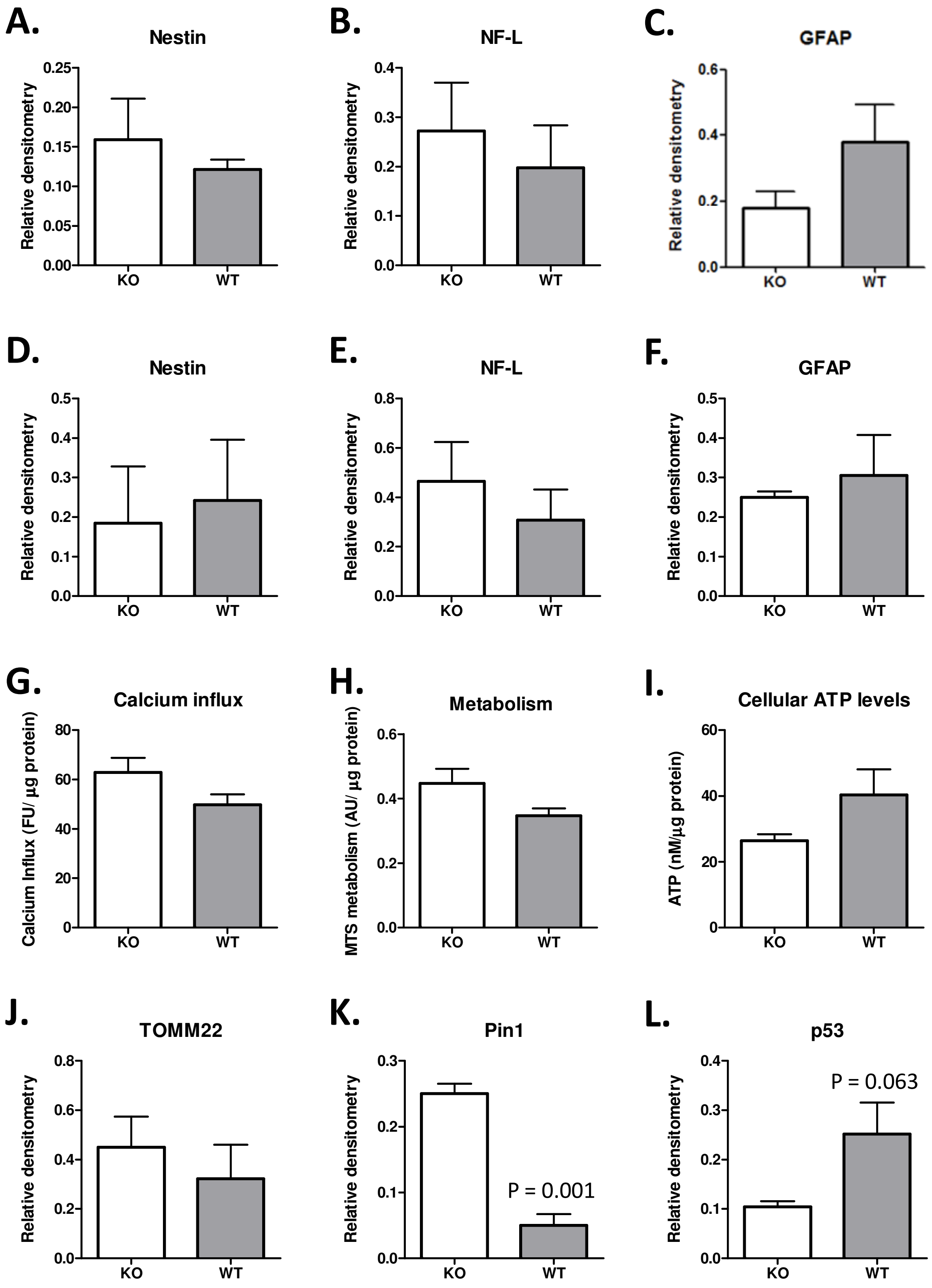
A.
Nestin
Relative densitometry
0.25
0.20
0.15
0.10
0.05
0.00
KO
WT
B.
NF-L
Relative densitometry
0.4
0.3
0.2
0.1
0.0
KO
WT
C.
GFAP
Relative densitometry
0.6
0.4
0.2
0.0
KO
WT
D.
Nestin
Relative densitometry
0.5
0.4
0.3
0.2
0.1
0.0
KO
WT
E.
NF-L
Relative densitometry
0.8
0.6
0.4
0.2
0.0
KO
WT
F.
GFAP
Relative densitometry
0.5
0.4
0.3
0.2
0.1
0.0
KO
WT
G.
Calcium influx
Calcium Influx (FU/ µg protein)
80
60
40
20
0
KO
WT
H.
Metabolism
MTS metabolism (AU/ µg protein)
0.6
0.4
0.2
0.0
KO
WT
I.
Cellular ATP levels
ATP (nM/µg protein)
60
40
20
0
KO
WT
J.
TOMM22
Relative densitometry
0.8
0.6
0.4
0.2
0.0
KO
WT
K.
Pin1
Relative densitometry
0.3
0.2
0.1
0.0
P = 0.001
KO
WT
L.
p53
Relative densitometry
0.4
0.3
0.2
0.1
0.0
P = 0.063
KO
WT
