# Supplementary material for: The prion protein regulates beta-amyloid-mediated self-renewal of neural stem cells in vitro
Source: Stem Cell Res Ther. 2015 Apr 11;6(1):60. doi: 10.1186/s13287-015-0067-4 (PMC4435829; doi:10.1186/s13287-015-0067-4)

**Additional file 5: PrP expression level changes in WT cells treated with Aβ.**

Quantification of PrP detected in WT NSCs treated with Aβ for 24 hours during proliferation (**A**) or for 7 days during differentiation (**B**). Quantifications are expressed as band densitometry relative to the coomassie protein stain for protein loading. No statistically significant differences in PrP expression were measured. N = 3.

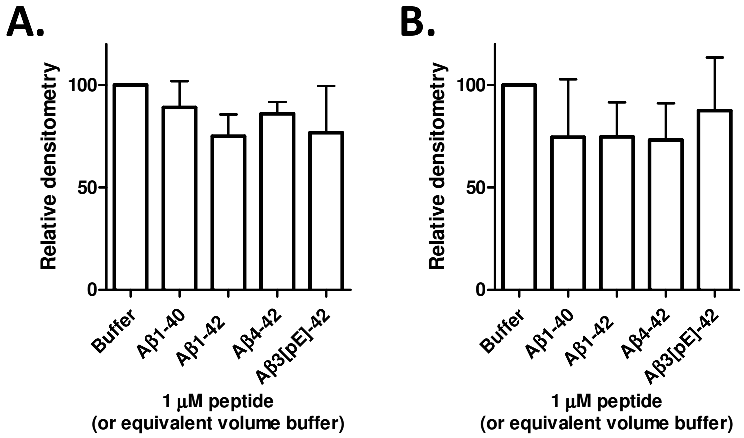

Supplement: Additional file 5: — Baseline comparisons of KO and WT cells. [file 13287_2015_67_MOESM5_ESM.pdf]
