# Supplementary material for: The prion protein regulates beta-amyloid-mediated self-renewal of neural stem cells in vitro
Source: Stem Cell Res Ther. 2015 Apr 11;6(1):60. doi: 10.1186/s13287-015-0067-4 (PMC4435829; doi:10.1186/s13287-015-0067-4)
Supplement: Additional file 6: — PrP expression level changes in WT cells treated with Aβ. [file 13287_2015_67_MOESM6_ESM.pdf]

Additional file 6: Further signalling pathways examined in cells treated with Aβ.

Previous studies have shown that Aβ signalling through PrP activates fyn and GSK3β and induces calcium signalling [1]. **A**. Phosphorylation, as an indication of activation, was determined for fyn and GSK3β (denoted pfyn and pGSK3β) by western blotting. NSCs were exposed to the Aβ peptides for 15 minutes in proliferation media, a time point previously determined to show significant changes in signalling molecule phosphorylation upon stimulation [2]. No significant changes in phosphorylation of either signalling molecule were observed. This is consistent with previous findings that GSK3β and tau do not influence neurogenesis in the adult organism [3]. **B**. Changes in calcium flux were assessed after 24 hours exposure to Aβ using the below protocol. No PrP specific changes were observed but an increased calcium influx was measured in cells treated with Aβ1-40 and in WT cells treated with Aβ1-42, which was not significantly different to KO cell responses (statistical analyses are shown in Sup Table ; *$p<0.05$, **$p<0.01$). This Aβ species-specificity suggest calcium signalling is unlikely to be involved in the KO or WT growth changes, which were PrP but not Aβ species-specific.

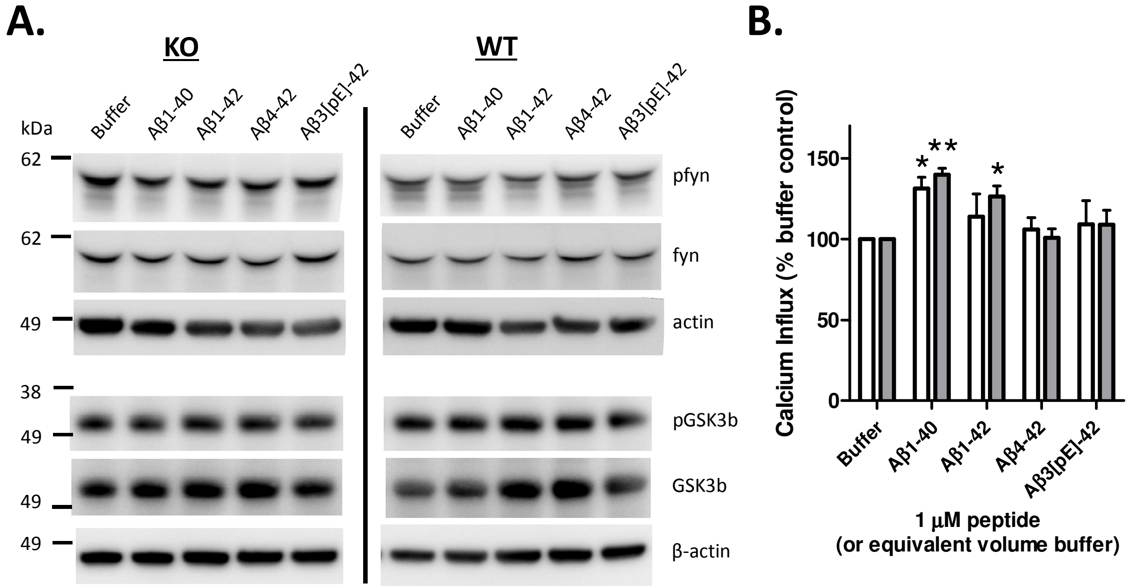

Calcium assay

Cells were seeded in 96-well plates as above. At the beginning of the assay 50 µl of culture media was removed from each we and replaced with calcium assay working buffer as per

manufacturer's instructions (Invitrogen). Plates were incubated for one hour under normal incubator conditions protected from light before readings were taken using 488 nm excitation and 530 nm emission filters in a FluoSTAR Optima (BMG labtech).

1. Lee IS, Jung K, Kim IS, Park KI: **Amyloid-beta oligomers regulate the properties of human neural stem cells through GSK-3beta signaling.** *Exp Mol Med* 2013, **45:**e60.
2. Haigh CL, Lewis VA, Vella LJ, Masters CL, Hill AF, Lawson VA, Collins SJ: **PrPC-related signal transduction is influenced by copper, membrane integrity and the alpha cleavage site.** *Cell Res* 2009, **19:**1062-1078.
3. Hong XP, Peng CX, Wei W, Tian Q, Liu YH, Cao FY, Wang Q, Wang JZ: **Relationship of adult neurogenesis with tau phosphorylation and GSK-3beta activity in subventricular zone.** *Neurochem Res* 2011, **36:**288-296.
