# Supplementary material for: The prion protein regulates beta-amyloid-mediated self-renewal of neural stem cells in vitro
Source: Stem Cell Res Ther. 2015 Apr 11;6(1):60. doi: 10.1186/s13287-015-0067-4 (PMC4435829; doi:10.1186/s13287-015-0067-4)
Supplement: Additional file 7: — Statistical analyses of data presented in Figure 3 . [file 13287_2015_67_MOESM7_ESM.pdf]

Additional file 7: Statistical analyses of Aβ data presented in Figure 1 and additional file 2.

ns = not significant, TG = Tga20.

**Test**	**Comparison**	**Aβ1-40**	**Aβ1-42**	**Aβ4-42**	**Aβ3[pE]-42**
Neurosphere volume - Fig 1B	KO Aβ vs buffer	P < 0.05	P < 0.05	ns	P < 0.001
2-way ANOVA, F = 53.75, p <	WT Aβ vs buffer	P < 0.01	P < 0.05	P < 0.05	ns
0.001, n = 4	KO vs WT	P < 0.001	P < 0.001	P < 0.01	P < 0.001
	TG Aβ vs Buffer	ns	ns	ns	P < 0.05
	KO vs TG	P < 0.001	P < 0.001	P < 0.05	P < 0.001
	WT vs TG	Ns	ns	ns	ns
Neurosphere diameter - Fig 1C	KO Aβ vs buffer	ns	P < 0.05	ns	P < 0.01
2-way ANOVA, F = 63.43, p <	WT Aβ vs buffer	P < 0.01	P < 0.001	P < 0.01	P < 0.05
0.001, n = 4	KO vs WT	P < 0.001	P < 0.001	P < 0.01	P < 0.001
	TG Aβ vs Buffer	P < 0.001	P < 0.001	P < 0.001	P < 0.001
	KO vs TG	P < 0.001	P < 0.001	P < 0.001	P < 0.001
	WT vs TG	Ns	ns	ns	ns
Neurosphere colonies - Fig 1D	KO Aβ vs buffer	ns	ns	ns	P < 0.05
2-way ANOVA, F = 9.647, p <	WT Aβ vs buffer	ns	P < 0.05	ns	ns
0.001, n = 4	KO vs WT	ns	ns	ns	ns
	TG Aβ vs Buffer	P < 0.05	P < 0.001	P < 0.001	P < 0.05
	KO vs TG	ns	ns	P < 0.001	P < 0.05
	WT vs TG	ns	ns	P < 0.01	ns
Cell cycle G0/G1 phase - Fig 1F	KO Aβ vs buffer	--	P < 0.05	--	--
2-way ANOVA, F = 22.99, p =	WT Aβ vs buffer	--	ns	--	--
0.003, n = 4	KO vs WT	--	P < 0.05	--	--
Cell cycle S phase - Fig 1F	KO Aβ vs buffer	--	ns	--	--
2-way ANOVA, F = 39.18, p =	WT Aβ vs buffer	--	ns	--	--
0.0033, n = 4	KO vs WT	--	P < 0.01	--	--

Cell cycle G2/M phase - Fig 1F	KO Aβ vs buffer	--	$P < 0.05$	--	--
2-way ANOVA, $F = 24.91$, $p = 0.0025$, $n = 4$	WT Aβ vs buffer	--	ns	--	--
	KO vs WT	--	$P < 0.01$	--	--

**Test**	**Comparison**	**Result**
Neurosphere volume	KO vs WT (untreated)	$P < 0.05$
2-way ANOVA, $F = 7.48$, $p = 0.006$, $n = 4$	KO vs TG (untreated)	$P < 0.05$
	WT vs TG (untreated)	ns
	KO untreated vs buffer	ns
	WT untreated vs buffer	ns
	TG untreated vs buffer	ns
Neurosphere diameter	KO vs WT (untreated)	$P < 0.05$
2-way ANOVA, $F = 10.01$, $p = 0.003$, $n = 4$	KO vs TG (untreated)	ns
	WT vs TG (untreated)	ns
	KO untreated vs buffer	ns
	WT untreated vs buffer	ns
	TG untreated vs buffer	ns
Neurosphere colonies	KO vs WT (untreated)	$P < 0.01$
2-way ANOVA, $F = 22.13$, $p < 0.001$, $n = 4$	KO vs TG (untreated)	ns
	WT vs TG (untreated)	$P < 0.001$
	KO untreated vs buffer	ns
	WT untreated vs buffer	ns
	TG untreated vs buffer	ns
