# Supplementary material for: The prion protein regulates beta-amyloid-mediated self-renewal of neural stem cells in vitro
Source: Stem Cell Res Ther. 2015 Apr 11;6(1):60. doi: 10.1186/s13287-015-0067-4 (PMC4435829; doi:10.1186/s13287-015-0067-4)
Supplement: Additional file 8: — Further signalling pathways examined in cells treated with Aβ. [file 13287_2015_67_MOESM8_ESM.docx]

**Additional file 8: Statistical analyses of data presented in Figure 3 and additional file 4.**

ns = not significant.

| **Test** | **Comparison** | **Aβ1-40** | **Aβ1-42** | **Aβ4-42** | **Aβ3[pE]-42** |
| --- | --- | --- | --- | --- | --- |
| MTS metabolism - Fig 3B  2-way ANOVA, F = 16.55, p < 0.001, n = 3 | KO Aβ vs buffer  WT Aβ vs buffer  KO vs WT | P < 0.05  ns  P < 0.05 | ns  ns  ns | ns  ns  P < 0.05 | ns  ns  ns |
| Cellular ATP - Fig 3C  2-way ANOVA, F = 5.95, p = 0.021, n = 4 | KO Aβ vs buffer  WT Aβ vs buffer  KO vs WT | ns  ns  ns | ns  ns  ns | ns  P < 0.05  ns | ns  ns  ns |
| TOMM22 densitometry - Fig 3E  2-way ANOVA, F = 16.45, p < 0.001, n = 3 | KO Aβ vs buffer  WT Aβ vs buffer  KO vs WT | ns  P < 0.05  ns | ns  ns  ns | ns  P < 0.05  P < 0.01 | P < 0.01  ns  P < 0.01 |
| Pin1 densitometry - Fig 3G  2-way ANOVA, F = 21.98, p < 0.001, n = 4 | KO Aβ vs buffer  WT Aβ vs buffer  KO vs WT | ns  ns  ns | ns  ns  ns | ns  ns  p < 0.05 | ns  ns  ns |
| p53 densitometry - Fig 3H  2-way ANOVA, F = 0.702, p = 0.412, n = 4 | KO Aβ vs buffer  WT Aβ vs buffer  KO vs WT | ns  ns  ns | ns  ns  ns | ns  ns  ns | ns  ns  ns |
| PrP densitometry - proliferating cells (24h) - S3 | WT Aβ vs buffer | ns | ns | ns | ns |
| PrP densitometry - differentiated cells (7d) - S3 | WT Aβ vs buffer | ns | ns | ns | ns |
| Calcium influx - S3  2-way ANOVA, F = 7.05, p = 0.002, n = 4 | KO Aβ vs buffer  WT Aβ vs buffer  KO vs WT | P < 0.05  P < 0.01  ns | ns  P < 0.05  ns | ns  ns  ns | ns  ns  ns |
